# HIV-1 low-level viraemia assessed with 3 commercial real-time PCR assays show high variability

**DOI:** 10.1186/1471-2334-12-100

**Published:** 2012-04-24

**Authors:** Jean Ruelle, Laurent Debaisieux, Ellen Vancutsem, Annelies De Bel, Marie-Luce Delforge, Denis Piérard, Patrick Goubau

**Affiliations:** 1 UCLouvain, AIDS Reference Laboratory, Avenue Hippocrate 54 - B1.54.05, 1200 Brussels, Belgium; 2 Hpital Universitaire Erasme, AIDS Reference Laboratory, Route de Lennik 808, 1070 Brussels, Belgium; 3 Vrije Universiteit Brussel, AIDS Reference Laboratory, site Universitair Ziekenhuis Brussel, Laarbeeklaan 101, 1090 Brussels, Belgium

**Keywords:** Residual viraemia, HIV-1 RNA, Viral load, Assay variability, Blip

## Abstract

**Background:**

Current real-time PCR-based HIV-1 viral load (VL) assays allow the detection of residual viraemia in antiretroviral-treated patients. The clinical outcome of HIV1 patients experiencing low-level replication (<50 cop/mL) in comparison with fully suppressed patients is currently debated. We analysed variability of 3 VL assays <50 cop/mL, and evaluated the reproducibility of viral blips <100 cop/mL.

**Methods:**

Three commercial VL assays were tested: Versant HIV-1 RNA 1.0 kPCR (Siemens), Abbott Realtime HIV-1, and Cobas Ampliprep/Cobas Taqman HIV-1 v2.0 (Roche). Ten replicates of a reference sample at 4 low target dilutions were tested to evaluate assay variability. Prospective collection of 181 clinical samples with detectable VL <50 cop/mL was used to evaluate intra-and inter-assay variability by triplicate testing. Samples from 26 patients experiencing a viral blip were retested.

**Results:**

All assays showed substantial variability at low VL level: the coefficient of variation at 100, 50, 25 and 12 cop/mL ranged respectively from 32 to 44%, 35 to 68%, 41 to 83% and 33 to 77%. In the intra-assay evaluation of repeatability, 52.5 to 57.5% of detectable VL <50 cop/mL tested in triplicate showed at least one fully undetected result. Variability was similar in the inter-assay arm. The VL blips could only be reproduced in 19% of cases.

**Conclusions:**

The most recent versions of widespread commercial VL assays showed substantial variability at low levels and residual viraemia could not be consistently reproduced. Patient outcome studies comparing residual VL to full suppression are therefore biased when using commercial assays.

## Background

Plasma viral load (VL) measurements are part of the routine follow-up for patients infected with HIV [1,2]. Antiretroviral therapy (ART) aims to block viral replication, leading to plasma viraemia below the clinically validated threshold of 50 copies per millilitre (cop/mL) of plasma. Below this limit, the patient has the lowest morbidity and mortality probabilities [3]. However, this threshold of 50 cop/mL reflects the performance characteristics of VL assays available before the launch of real-time PCR-based assays, which allow the detection of low numbers of RNA copies present in the sample.

The limit of detection (LoD) does not usually equal the lower limit of quantification (LLoQ) at which the error meets the requirements for reproducibility and linearity [4]. Under the linear range, variability is unacceptably high and accurate quantification remains elusive [5,6]. Nonetheless, recent VL assays report qualitative results below LLoQ as detected or not detected, suggesting the presence or absence of residual viraemia.

The cause and the consequence of low VL are currently investigated [7]: HIV RNA can originate from the release of virus from the reservoirs or from residual replication [8]. Using modified protocols of commercial assays or in-house assays to monitor VL, intensification of ART in patients with incompletely suppressed VL bore no result [9], and low-level viraemia was not associated with suboptimal CD4 gains during therapy [10]. Comparisons of drug efficacy on residual viraemia showed favourable profile for fixed-dose combinations including efavirenz [11], although others showed superior outcome when using nevirapine instead of efavirenz [12]. Some studies suggest that residual viraemia is predictive of future treatment failure [13], whilst some suggest no further effect [14]. We hypothesised that those results could not be translated to routine settings because they exceed the technical abilities of current commercial real-time PCR assays. We evaluated the variability of 3 commercial real-time HIV-1 VL assays on samples with detectable VL but <50 cop/mL, and analysed the repeatability of low-level viraemia in clinical routine settings in order to demonstrate the hypothesis.

## Results

### Dilutions of a reference sample

The descriptive statistics and the number of undetected samples are illustrated in Table 1. No signal was detected for any of the HIV-negative samples or negative kit controls throughout the study with any of the assays.

**Table 1:** Descriptive statistics related to 10 replicates of a reference sample at four target dilutions

		**Siemens**	**Abbott**	**Roche**
100 cop/ml	Mean (cop/mL)	60.6	84.5	115.4
	SD (cop/mL)	26.7	27.7	38.3
	CV (%)	44.0	32.8	33.2
	Undetected (%)	0	0	0
50 cop/ml	Mean (cop/mL)	35.3	33	65.8
	SD (cop/mL)	15.3	22.5	22.7
	CV (%)	43.2	68.1	34.5
	Undetected (%)	0	0	0
25 cop/ml	Mean (cop/mL)	18.1	25.5	26.56
	SD (cop/mL)	8.7	10.4	22.1
	CV (%)	48.1	40.7	83.1
	Undetected (%)	0	0	10
12 cop/ml	Mean (cop/mL)	15.6	20.0	25.8
	SD (cop/mL)	6.6	6.6	19.9
	CV (%)	42.2	32.8	77.1
	Undetected (%)	20	70	20

Undetected samples were censored from the calculations. Siemens=Versant HIV-1 RNA 1.0 kPCR (Siemens), Abbott=Realtime HIV-1 (Abbott), Roche=Cobas Ampliprep/Cobas Taqman HIV-1 v2.0 (Roche), SD: standard deviation, CV: coefficient of variation.

**Figure 1:**
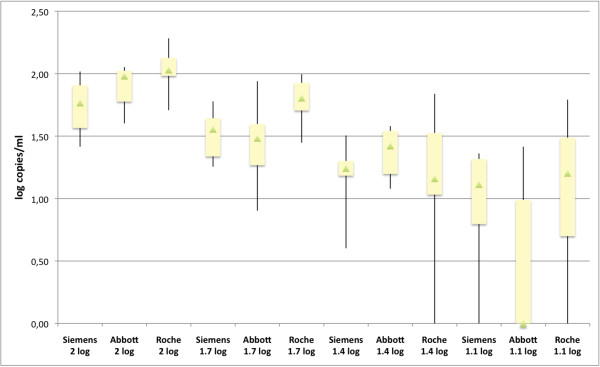
**Low-level VL variability evaluated with a reference sample.** Box Plot showing the distribution of 10 replicates of a reference sample at four dilutions (100, 50, 25 and 12 cop/ml or 2, 1.7, 1.4 and 1.1 log cop/ml) tested on each platform. The bottom and the top of the box represent the lower and the upper quartiles respectively. The triangle in the box is the median, and the ends of the whiskers correspond to the minimum and the maximum values. Siemens=Versant HIV-1 RNA 1.0 kPCR (Siemens), Abbott=Realtime HIV-1 (Abbott), Roche=Cobas Ampliprep/Cobas Taqman HIV-1 v2.0 (Roche).

As noted during the preparation of the reference sample, the absolute quantification was the highest for the CAP/CTM v2.0, and the lowest for the kPCR that qualified 30% of the 100 cop/mL samples as detected below its LLoQ. At the target dilution of 12 cop/mL, the Abbott Realtime HIV-1 assay showed the highest rate of undetected samples with 70% of negative results while the others had 20% in comparison. Variability was the highest for CAP/CTM v2.0: the CV ranged from 33 to 83%, while the CV of kPCR and Realtime assays varied respectively from 42 to 48%, and from 32 to 68%. Of note, CAP/CTM v2.0 has the highest variability at the 25 cop/mL target and failed to detect one sample at a value theoretically above its LLoQ of 20, although below the LLoQ of the comparators. The distribution of values at each concentration is shown on Figure 1. Based on this set of results, the LoDs inferred by Pobit analysis were 14.76, 18.89 and 23.19 cop/mL respectively for the kPCR, Abbott realtime and CAP/CTM v2.0, limit defined as the RNA concentration detected with 95% or greater probability.

### Clinical samples

The variability was tested on 181 clinical samples obtained from patients experiencing residual viraemia <50 cop/mL. As samples had been selected prospectively and consecutively, with the only criterion defined as a detectable VL <50 cop/mL, we checked if the viral subtype was representative for our population. A genotype was available for 61.2% of the patients. The clade prevalence in our cohort was similar to the Belgian HIV-1 population, the most abundant ones being subtype B, CRF02, A1 and C: respectively 47, 14, 14 and 10% of the patients.

**Figure 2:**
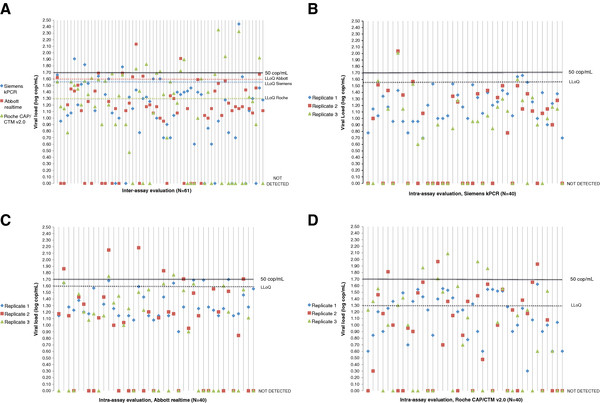
**Repeatability of VL below 50 cop/ml in clinical settings. A**. Inter-assay variability. The plasma VL of sixty-one samples were tested with the assays from Siemens=Versant HIV-1 RNA 1.0 kPCR (Siemens), Abbott=Realtime HIV-1 (Abbott), Roche=Cobas Ampliprep/Cobas Taqman HIV-1 v2.0 (Roche). **B**. Intra-assay repeatability on 40 triplicates tested with Versant HIV-1 RNA 1.0 kPCR (Siemens). **C**. Intra-assay repeatability on 40 triplicates tested with Realtime HIV-1 (Abbott). **D**. Intra-assay repeatability on 40 triplicates tested with Cobas Ampliprep/Cobas Taqman HIV-1 v2.0 (Roche).

Table 2 details the number of samples with discordant results between triplicates. All assays behaved similarly to over 50% of samples where at least one replicate was not detected. Between 2.5 and 5% of the samples had one replicate >100 cop/mL. Figure 2 details the distribution of the 61 samples tested by inter-assay comparison, as well as the distribution of 40 triplicates for each method in the intra-assay evaluation: variability is the cause of frequent results above the threshold of 50 cop/mL, as well as undetected ones.

**Table 2:** Intra- and inter-assay evaluation of repeatability with clinical samples from patients experiencing residual viraemia

	**% of samples where at least one replicate is not detected**	**% of samples where only one triplicate is detected**	**% of samples where at least one replicate was higher than 50 cop/mL**	**% of samples where at least one replicate was higher than 100 cop/mL**	**% of samples where at least one replicate was higher than 200 cop/mL**
Intra-assay Siemens (N=40)	52.5	32.5	2.5	2.5	0.0
Intra-assay Abbott (N=40)	57.5	20.0	15.0	5.0	0.0
Intra-assay Roche (N=40)	55.0	20.0	12.5	2.5	0.0
Inter-assay (N=61)	52.5	13.1	18.0	4.9	3.3

Each sample was tested in triplicate, the first replicate being detected but quantified below 50 cop/mL. Siemens=Versant HIV-1 RNA 1.0 kPCR (Siemens), Abbott=Realtime HIV-1 (Abbott), Roche=Cobas Ampliprep/Cobas Taqman HIV-1 v2.0 (Roche).

### Viral blips

Medical records were examined and 26 cases corresponding to our blip definition (between 50 and 100 cop/mL) were re-analysed. Patients experiencing those blips represented between 1 and 1.5% of the population followed in the participating centres. The VL sequence of the blips, i.e. a VL <50 cop/mL followed by a value between 50 and 100 cop/mL going back afterwards <50, could only be reproduced in 5 cases (19.2%). In the remaining 21 cases, 16 were detected below 50 cop/mL and 5 were not detected. All samples preceding and following the putative blip gave a <50 cop/mL result again, except for one case where the value before the blip and the blip itself were respectively 62 and 64 cop/mL.

## Discussion

Variability of three commercial HIV-1 VL assays was investigated at low values below the clinical cut-off of 50 cop/mL.

As all the assays tested used automated platforms for viral RNA extraction and PCR reactive handling, no discrepancies due to human interference are to be considered. Moreover, the samples underwent no additional thaw and freeze cycle. Depending on the assay, variability at 50 cop/mL rose from 34 to 68% (CV) when a reference sample was diluted in human HIV-negative plasma: results round that cut-off are therefore uncertain, making a precise estimation of values just above 50 cop/mL difficult. Even at the 100 cop/mL target, 30% of samples were quantified<LLoQ with the kPCR assay. When focussing on lower values, variability was the highest with the CAP/CTM v2.0 assay. The latter missed 1 out of 10 replicates above its LLoQ. The Abbott Realtime HIV-1 assay seemed less sensitive than the others at the 12 cop/mL level, although the LoDs inferred from our results are similar with the 3 assays, ranging from 15 to 23 cop/mL.

Repeatability of RNA detection <50 cop/mL was evaluated on 181 triplicates, either intra- or inter-assay: more than 50% of results cannot be reproduced on the 3 aliquots.

Those observations are in line with the PCR limits, as well as with the limits given by the assay manufacturers: every PCR will lead to stochastic results when targets introduced in the reaction mixture lie between the limit of blank and the limit of detection [4], and companies define their own assay lowest LLoQ level below which the results cannot be treated in a quantitative manner. Extreme values cannot be reproduced quantitatively and are therefore considered as lower than LLoQ in clinical practice.

The results below the LLoQ are reported as detected or not detected by the assays software, and such information should not be reported to the clinician because of uncertainty. This distinction between the presence and absence of PCR signal, together with the launch of assays with LoQ below 50 cop/mL, led to the notion that some patients previously considered as therapy-controlled undergo low-level viraemia, and that others are fully suppressed, i.e. no viral genome can be detected in the plasma. Studies investigating patient outcome when residual viraemia is detected were published, as well as intensification trials exploring the effect of an additional antiretroviral drug on full viral suppression. Some authors used methodology enabling to concentrate the HIV-1 RNA by extracting from 4mL up to 30mL of plasma following ultracentrifugation [15-17], or repeat the test 3 times [18], lowering the detection limits of PCR and reducing the variability in the low copy number range. Those modified assays may be useful to answer scientific questions about low-level viraemia, but cannot be translated into clinical practice because the variability of most recent versions of widespread commercial VL assays is too high.

Our study was not designed to study the assays specificities. Previous studies showed that HIV-negative samples do not lead to false positive signal, as confirmed here with 10 negative samples. Low values can indeed be considered as true viraemia [19] but negative ones cannot be considered as proof of full viral suppression. RNA viral genomes can originate from long-living infected cells producing viral particles in the absence of replication, or reflect a residual replication. Strategies aiming at viral eradication need to target the source of circulating viruses [20]. Whether this residual viraemia is the beginning of virological failure is subject to debate [7,13,14]. We would suggest that current commercial real-time PCR assays do not have sufficient precision to answer this burning question. Variability in the low copy number range has also implications both for the classification of elite controllers as well as for diagnosis strategies using molecular assays.

In the same way, outcome comparisons of different ART regimens regarding full suppression must be powered enough to take into account assay variability. Although the samples used in this study were taken from patients on suppressive ART, the population was not big enough to detail the drug classes used in the subgroup of reproducible low VL versus the others.

Viral blips between 50 and 100 cop/mL were not reproduced in 80% of the 26 cases. As analytical assay variability is high around 50 cop/mL (Figure 1), it is therefore useful in clinical practice to confirm the result on a new sample to assess if viral replication is really increasing: the samples following the blips tested here were all repeatedly <50 cop/mL. Our results are in line with the 2011 revision of the DHHS guidelines which define the virological failure threshold as 200 cop/mL [1]. Consequently, a single value between 50 and 200 cop/mL should not be a reason enough for dropping a case out of clinical studies.

## Conclusions

In conclusion, state of the art VL assays tested here showed poor reproducibility of HIV-1 low-level viraemia results. When used in clinical routine settings with single measurements, they introduce biases in patient outcome studies comparing very low VL to full viral suppression.

## Methods

### Participating laboratories and assays

Three AIDS reference laboratories in Brussels accredited by national legal bodies to either the EN-ISO 15189 or the EN-ISO 17025 standard performed the analyses, one assay running per site. The assays tested were Versant HIV-1 RNA 1.0 kPCR (Siemens, performed at UCLouvain, using 0.5mL of plasma), Realtime HIV-1 (Abbott, performed at Erasme using the 0.6mL plasma volume protocol), and Cobas Ampliprep/Cobas Taqman HIV-1 v2.0 (CAP/CTM, Roche Diagnostics, perfomed at UZ Brussel, using 0.85mL of plasma). The manufacturers respectively report the LLoQs as 37, 40 and 20 cop/mL. The volumes of plasma mentioned are those actually extracted by the platforms, excluding the dead volume linked to the pipetting steps. Only a part of the RNA is introduced in the RT-PCR reaction: respectively 79%, 56% and 67% of the total elution volume, corresponding to 0.39, 0.33 and 0.57mL of plasma.

The manufacturers recommendations were strictly followed and the same reagent lot was used throughout the study, except for the re-evaluation of blips.

We did obtain quantitative results beyond the assays LLoQs using the Cq and the calibration curve and the manufacturing companies offered some help when the assay software did not produce a value in IVD conditions.

### Dilutions of a reference sample

A reference HIV-1 sample, i.e. a culture supernatant dilution of a subtype B isolate, is routinely used in all of the Belgian centres as an internal control in each VL run. We assigned a true value to that reference sample based on the results obtained in the last 25 VL runs per assay: the mean VL SD was 3.13 0.12 (Siemens), 3.18 0.07 (Abbott) and 3.37 0.08 log_10_ cop/mL (Roche). The average value of 3.23 log_10_ cop/mL was used to prepare aliquots of 100, 50, 25 and 12 cop/mL (2, 1.7, 1.4 and 1.1 log cop/mL) in commercial HIV-negative human plasma (A&E Scientific, Belgium). Participating laboratories blind tested 10 aliquots of each dilution as well as 10 aliquots of the negative plasma used to prepare the samples. The LoD was inferred by probit analysis using the IBM SPSS Statistics 20 software.

### Variability in patients with residual viraemia

The variability in clinical settings with patients experiencing residual viraemia below 50 cop/mL was assessed with 181 samples. Each laboratory selected prospectively and consecutively 62 plasma samples: when a fresh whole EDTA blood sample was received for HIV-1 VL testing, 3 aliquots were frozen and stored below 70C. If the first aliquot used for routine analysis gave a detectable result <50 cop/mL, the sample was included. Apart from this condition and the availability of sufficient sample volume, no selection for the inclusion of samples was made. Only one sample per patient was included. The maximal duration of storage was limited to 6weeks and no additional freeze/thaw cycles were allowed. Forty samples were tested in triplicate on the same platform during different runs to assess the intra-assay variability, and 22 others were shared with the 2 other laboratories for an inter-assay evaluation. If one replicate gave an invalid result, the sample was excluded from the analysis: 61 samples were finally analysed in the inter-assay evaluation whilst 5 were excluded because of failure of 1 replicate. The TRUGENE HIV-1 genotyping kit (Siemens, Tarrytown, NY) determined the HIV-1 subtype based on the reverse transcriptase sequence.

### Viral blips

Laboratories searched their records to identify patients who experienced a blip, i.e. a VL between 50 and 100 cop/mL during the follow-up and their previous and next sample was <50 cop/mL, during the last 15months. The median time between the first and the second analysis was 7months (ranging from 3 to 15), plasma samples being stored at 80C. Twenty-six blips, together with the previous and next sample from that patient, were retested with the same assay and without additional thaw and freeze cycle.

## Abbreviations

VL = Viral load; ART = antiretroviral therapy; cop/mL = copies/mL; LoD = limit of detection; LLoQ = lower limit of quantification; Cq = cycle of quantification; CAP/CTM = Cobas Ampliprep/Cobas Taqman HIV-1 v2.0; CV = coefficient of variation; IVD = in vitro diagnostic.

## Competing interests

JR was invited twice as a speaker for Siemens Healthcare Diagnostics. The other authors have declared no competing interests.

## Authors contributions

JR initiated the study, centralised the data and drafted the manuscript. LD, EV and ADB participated in the study design and helped to draft the manuscript. MLD, DP and PG revised the design of the draft and provided critical input. All authors read and approved the final manuscript.

## Pre-publication history

The pre-publication history for this paper can be accessed here:

http://www.biomedcentral.com/1471-2334/12/100/prepub
